# A Comparison of Neuroimaging Abnormalities in Multiple Sclerosis, Major Depression and Chronic Fatigue Syndrome (Myalgic Encephalomyelitis): is There a Common Cause?

**DOI:** 10.1007/s12035-017-0598-z

**Published:** 2017-05-17

**Authors:** Gerwyn Morris, Michael Berk, Basant K. Puri

**Affiliations:** 1 Tir Na Nog, Bryn Road Seaside 87, Llanelli, Wales SA15 2LW UK; 2 The Centre for Molecular and Medical Research, School of Medicine, Deakin University, P.O. Box 291, Geelong, 3220 Australia; 3 Department of Clinical Medicine and Translational Psychiatry Research Group, Faculty of Medicine, Federal University of Ceará, Fortaleza, CE 60430-040 Brazil; 4 IMPACT Strategic Research Centre, School of Medicine, Deakin University, P.O. Box 291, Geelong, 3220 Australia; 5 Orygen Youth Health Research Centre and the Centre of Youth Mental Health, The Florey Institute for Neuroscience and Mental Health and the Department of Psychiatry, University of Melbourne, Parkville, 3052 Australia; 6 Department of Medicine, Imperial College London, Hammersmith Hospital, London, England W12 0HS UK

**Keywords:** Chronic fatigue syndrome, Depression, Inflammation, Multiple sclerosis, Neuroimaging

## Abstract

There is copious evidence of abnormalities in resting-state functional network connectivity states, grey and white matter pathology and impaired cerebral perfusion in patients afforded a diagnosis of multiple sclerosis, major depression or chronic fatigue syndrome (CFS) (myalgic encephalomyelitis). Systemic inflammation may well be a major element explaining such findings. Inter-patient and inter-illness variations in neuroimaging findings may arise at least in part from regional genetic, epigenetic and environmental variations in the functions of microglia and astrocytes. Regional differences in neuronal resistance to oxidative and inflammatory insults and in the performance of antioxidant defences in the central nervous system may also play a role. Importantly, replicated experimental findings suggest that the use of high-resolution SPECT imaging may have the capacity to differentiate patients afforded a diagnosis of CFS from those with a diagnosis of depression. Further research involving this form of neuroimaging appears warranted in an attempt to overcome the problem of aetiologically heterogeneous cohorts which probably explain conflicting findings produced by investigative teams active in this field. However, the ionising radiation and relative lack of sensitivity involved probably preclude its use as a routine diagnostic tool.

## Introduction

Many patients with multiple sclerosis (MS), a neuroinflammatory illness, major depressive disorder (MDD), a neuroprogressive condition and chronic fatigue syndrome (CFS) (myalgic encephalomyelitis), a phenotype of no fixed aetiology, share a range of underlying abnormalities [1, 2]. These include mitochondrial dysfunction; systemic inflammation; oxidative stress; a range of autoimmune phenomena; hypothalamic-pituitary axis dysfunction and evidence of structural and functional brain change obtained by neuroimaging using magnetic resonance imaging (MRI), functional MRI (fMRI), magnetic resonance spectroscopy (MRS), positron emission tomography (PET) and single-photon emission computerised tomography (SPECT) (also known as single-photon emission tomography (SPET)) and cognitive symptoms [1–3]. Neuroimaging abnormalities in MS are well recognised, and there are numerous excellent reviews on the subject [4–6]. There are also many excellent reviews of neuroimaging abnormalities in MDD utilising voxel-based morphometry (VBM) which is widely used within neuropsychiatry [7–11]. Such neuroimaging abnormalities are routinely seen in many patients afforded a diagnosis of CFS according to international consensus criteria [3]. Such abnormalities have been discerned via MRI [12–14], fMRI [15–17], MRS [17–19] and PET [20].

Despite the existence of such data, however, there would appear to be no comprehensive review of neuroimaging abnormalities in patients afforded such a diagnosis which includes the most recent evidence obtained via VBM analysis. There have been few attempts to compare the transdiagnostic neuroimaging data obtained for patients with MS and MDD [2] although there have been several studies comparing neuroimaging abnormalities seen in MS patients with or without co-morbid depressive symptoms which appear to demonstrate changes in global and regional patterns of white and grey matter reduction [21–23]. Similarly, there have been few attempts to compare neuroimaging data in MS, MDD and CFS [2, 3]. We aim to remedy this by engaging in such an in-depth review. We have chosen to focus on data examining resting-state functional connectivity, white and grey matter structure and volume and cerebral perfusion for two main reasons. First, this renders the analysis manageable in terms of length. Second, and perhaps more importantly, these parameters are strongly influenced by the presence of systemic inflammation [24–31]. Hence, by comparing and contrasting neuroimaging data in these domains, we may be in a position to comment on mechanisms driving neuroimaging abnormalities in these illnesses which might be similar or different, which in turn could speak to differences in the pathogenesis of these conditions. To this end, we will review data on resting-state functional connectivity, white and grey matter abnormalities and abnormalities in cerebral perfusion in patients with various subtypes of MS before examining data relating to the same parameters in MDD and CFS.

## Neuroimaging Abnormalities in MS

### Resting-State Functional Connectivity in MS

Functional dissociation or disconnection between large-scale resting-state neural networks is a characteristic feature of relapsing-remitting MS (RRMS) in the advanced stage of the disease [32, 33]. Moreover, the degree of dissociation correlates with the severity of disability in multiple dimensions and with the extent of T2-lesion load [32, 33]. The clinical significance of these abnormalities is emphasised by data demonstrating that functional connectivity between networks is significantly stronger in patients with preserved cognitive function and correlates with lower lesion load and other structural abnormalities [34].

Cortical reorganisation in patterns of resting-state network connectivity is evident in patients with a clinically isolated syndrome [35] and is typically apparent as increased functional connectivity which is widely regarded as an adaptive modification in an attempt to mitigate the deleterious effects on brain function by disease processes [35, 36]. This pattern of reorganisation involves several networks and has been most commonly reported in the executive control network, sensory-mode network and default-mode network (DMN) [32, 37].

Some authors report an increase in the functional connectivity within resting-state networks in patients with RRMS [38–40] while others report a decrease [32, 41]. Specific resting networks such as the DMN and resting-state motor networks display increased or decreased connectivity with some other networks, which suggests a general reorganisation of connectivity between and within neural networks rather than a simple pattern of increase or decrease [39, 41].

Several research teams have also reported correlations between abnormal intra-network functional connectivity and clinical parameters, but these relationships are far from straightforward [42–44]. For example, while more studies report an inverse relationship between functional connectivity and clinical disability [45–47], other studies indicate a positive association between functional connectivity and clinical disability [39].

The same variation in patterns persists for specified dimensions of disability such as impaired motor function where reduced and increased connectivity within the network is associated with impaired motor performance [44, 48]. Increased connectivity within the dorsal premotor cortex correlates with increasing clinical disability as measured by the Expanded Disability Status Scale (EDSS), and this has been held once again to be an adaptive reorganisation aimed at preserving motor function [43]. However, this team of authors have also reported that impaired regional connectivity in the left cerebellar hemisphere, a region of the brain which regulates multiple aspects of motor performance, is associated with increasing clinical disability [39]. The latter pattern appears to predominate in advanced stages of disease where impaired connectivity throughout the motor network is associated with a higher burden of symptoms [48]. In this context, it is noteworthy that recovery from acute relapse appears to be associated with changes in resting-state motor network connectivity [49]. It has also been proposed that reduced activity in the medial prefrontal cortex or the anterior cingulate cortex (ACC) may in fact function as a state marker for the disease [50].

The picture is if anything more complex in studies investigating the resting-state functional connectivity associated with cognitive dysfunction within the DMN, as impaired and enhanced conductivity between and within sub-regions in the DMN have been repeatedly observed [51, 52]. However, once again, decreased connectivity within this region of the brain and associated impairment of cognitive performance appears to be the most common pattern with advancing disease severity [42, 47].

When considered as a whole, the data would suggest that in early or mild disease, there is a compensatory positive reorganisation within and between resting-state networks which eventually are overcome by disease progression [35, 53, 54]. This would explain data illustrating changes in the patterns of connectivity with increasing disease severity but not how such patterns are modified by gender, which seems to be an issue worthy of research [55]. Finally, impaired neural connectivity between the hippocampus and the subgenual cingulate, parietal and prefrontal regions correlates with the severity of depression [54]. Interestingly, such defects in connectivity are provoked by activated microglia in the hippocampal region [54].

### White Matter Abnormalities in MS

Conventional structural MRI of MS patients is characterised by the existence of cortical demyelination and focal and diffuse white matter (WM) lesions [56–58]. Researchers have reported a plethora of variations in T1 and T2 signals which may well reflect considerable intra-patient variability in underlying lesion pathology [59, 60]. Several research teams using MR approaches such as magnetisation transfer ratio (MTR), magnetic resonance spectroscopy (MRS) and diffusion tensor imaging (DTI) have reported a wide range of abnormalities in seemingly normal-appearing WM (NAWM) [61–63]. These include changes in the diffusion properties of water, reduced MTR, abnormal T1 and T2 relaxation times and a range of metabolic abnormalities including reduced *N*-acetyl aspartate, increased glutamate/glutamine and reduced choline [64–71]. In addition, prolonged T1 and T2 times have also been reported in diffusely abnormal WM (DAWM), which is suggestive of decreased myelin density, axonal loss and chronic fibrillary gliosis [72, 73]. It is also noteworthy that NAWM abnormalities revealed by MTR histogram analysis worsen over the disease course when compared with age- and sex-matched healthy controls [74, 75].

### Grey Matter Abnormalities in MS

Grey matter (GM) pathology is evident in the hippocampus, basal ganglia, hypothalamus, cerebellum and spinal cord [76]. GM atrophy is seen in the earliest stage of the disease, and its extent is predictive of the development of cognitive disability, physical disability (as indexed by the EDSS) and overall disease prognosis (reviewed [77, 78]). It is also of interest that GM atrophy increases over time as the disease worsens, which is a phenomenon which is not apparent in WM atrophy [79]. While there is scant post-mortem evidence of an inflammatory origin of GM pathology [80], biopsies taken from patients at early disease stages reveal a different picture—there is ample evidence of T-cell infiltration, activated microglia and astrocytes together with other markers of chronic inflammation associated with cortical lesions and diffuse injury [81, 82].

The reasons for the discrepancy between in vivo and post-mortem studies are not clear but may stem from re-myelination processes which are far more active in GM than WM (reviewed [83]). However, it should be noted that there is evidence of neurodegenerative as well as inflammatory damage in the GM of RRMS patients and current debate centres on whether such neurodegeneration is independent of inflammation or has an independent origin [79, 84]. There is evidence suggesting that neurodegenerative damage is secondary to mitochondrial dysfunction and impaired ion channel activity [85, 86]. Such data are difficult to interpret however as several authors have concluded that mitochondrial dysfunction seen in the central nervous system of MS patients is in fact driven by chronic inflammation in general and oxidative bursts in particular [87, 88]. Moreover, an intriguing study conducted by Frischer and fellow workers established an inverse relation between neuronal integrity in the GM and the magnitude of inflammation rather suggesting that neurodegenerative processes are not independent of inflammatory drivers [87]. Once again, given the relationship between the development of systemic inflammation and neuroinflammation, it seems reasonable to conclude that chronic inflammation in the periphery makes a significant contribution to the development of central GM pathology.

### Cerebral Perfusion in MS

There is a considerable and accruing body of research demonstrating globally reduced cerebral perfusion and blood volume abnormalities in MS patients obtained via the use of SPECT [89]; PET [90] and, more recently, dynamic susceptibility contrast-enhanced perfusion MRI [91–93]. Interestingly, this state of chronically impaired cerebral blood flow (CBF) appears to exist in all subtypes of the illness [94, 95], develops in patients with a clinically isolated syndrome and in very early disease [93, 96] and would seem to affect NAWN before deep GM changes [93].

Several research teams have reported relationships between abnormal patterns of perfusion and various elements involved in the pathophysiology or pathogenesis of the illness [94, 97–99]. For example, Taghizadeh and colleagues examined the brains of 25 RRMS patients via 99mTc-labelled SPECT and reported a positive association of the degree of perfusional impairment between disease duration and disease severity indexed by the EDSS [97]. Interestingly, these authors also reported a significant increase in perfusion following 20 sessions of hyperbaric oxygen treatment but unfortunately, this improvement was not accompanied by any improvement in disease status as measured by any objective or subjective parameter [97]. Another team of authors utilising dynamic susceptibility contrast-perfusion MRI reported a significant decrease in cerebral hypoperfusion in 22 RRMS patients, compared with an equal number of sex-matched controls, whose extent correlated with the severity of their self-reported fatigue [98]. There is also considerable evidence associating reduced CBF in GM and WM with the development of cognitive dysfunction in multiple domains [100–103].

## Neuroimaging Abnormalities in MDD

### Resting-State Functional Connectivity in MDD

Several studies have revealed dysfunction of functional connectivity within or between large-scale functional resting-state neural networks such as the DMN in patients with MDD using resting-state fMRI [104]. Abnormalities in two large-scale neuronal networks—the frontoparietal central executive network (CEN) and the medial prefrontal-medial parietal DMN—are consistent findings in depression and potential therapeutic targets for transcranial magnetic stimulation [105]. Resting-state functional conductivity is also altered by some psychotropics efficacious in depression such as ketamine, lithium and antidepressants [106, 107].

Intra-network changes are exemplified by enhanced connectivity within the anterior DMN and abnormal connectivity between the anterior and posterior DMN. Inter-network abnormalities are evidenced by increased connectivity between the anterior DMN and salience network and diminished connectivity between the posterior DMN and the CEN (review [108]). MDD network research has largely focused on the DMN which is understandable given that this network incorporates the anterior cingulate and an extensive area of the medial prefrontal cortex extending into the orbitofrontal cortices considered to play a major if not dominant role in the pathophysiology of the illness [109]. Anterior regions of the DMN, particularly the medial prefrontal cortex, mediate self-referential processing while posterior regions are associated with the formation and retrieval of episodic memories (review [109]).

Dissociation of functional connectivity between the anterior and posterior areas of this region and dissociation between the DMN and other large-scale resting networks are characteristic of patients with MDD [110, 111]. Zhu and fellow workers reported that a pattern of dissociation between anterior and posterior functional connectivity in resting-state DMNs of first-episode, treatment-naïve young adults with MDD is evidenced by increased functional connectivity in anterior medial cortical regions (especially the medial prefrontal cortex and ACC) and decreased functional connectivity in posterior medial cortical regions (especially the posterior cingulate cortex/precuneus) in MDD patients compared with control subjects [110]. In the depressed group, the increased functional connectivity in the anterior medial cortex correlated positively with rumination score, while the decreased functional connectivity in the posterior medial cortex correlated negatively with over-general autobiographical memory test scores [110]. More recent data have established a relationship between the patterns of functional dissociation in connectivity between the precuneus (PN), a region within the DMN, and the clinical presentation and/or severity of MDD [111, 112]. For example, the magnitude of hyperconnectivity between the PN and the dorsal medial prefrontal/ACC region is associated with increased severity while reduced connectivity between the PN and fusiform areas is associated with the variable symptoms seen in the illness.

While alterations in brain connectivity centring on the DMN continue to be a focus of research, abnormal patterns of connectivity are also seen within and between the affective network and the cognitive control network (CCN) [113, 114]. In fact, several trait- and state-dependent abnormalities in patterns of functional connectivity have been reported in the cerebellum, ACC, lingual gyrus, dorsolateral prefrontal cortex (DLPFC), middle frontal gyrus, amygdala and insula [113].

Successful therapy is associated with changes in connectivity within the DMN and corticolimbic areas, and it is noteworthy that changes in the latter areas correlate with the degree of clinical improvement while changes in the DMN do not [114]. The success or otherwise of transcranial magnetic stimulation would also appear to depend on the positive modulation of functional connectivity in this instance both between and within the CEN and DMN [105].

### White Matter Abnormalities in MDD

DTI utilising tract-based spatial statistics (TBSS) [115] has revealed disrupted WM tracts and WM degeneration in the prefrontal cortex, the temporal cortex and limbic system, the uncinate fasciculus, the internal capsule and the superior longitudinal fasciculus (review [116, 117]). The geographical extent of these WM tract abnormalities has prompted some authors to describe MDD as a disconnection syndrome [118]. DTI studies have reported microstructural abnormalities, albeit of a somewhat different pattern, in WM integrity characterised by low fractional anisotropy (FA) values associated with MDD in elderly patients [119, 120], middle-aged patients [121], young treatment-naïve first-episode adolescent patients [122, 123] (review [124]) and even disease-free individuals with a familial vulnerability to the development of the illness [125, 126]. A recent meta-analysis conducted by Chen and fellow workers using TBSS concluded that there was consistent evidence of WM architecture and integrity disruption in the frontal and thalamic regions, and the anterior limb of the internal capsule and importantly that WM reduction in the corpus callosum is associated with more severe disease [127].

Interestingly, Le Win and others reported similar findings using a DTI TBSS-based approach in adolescents with MDD [128]. Lower FA values in the WM associated with the corpus callosum and the cortical-striatal-thalamic circuit were detected, as well as lower FA values in the superior and anterior corona radiata [128]. Very similar findings in adolescent MDD have been reported by Hendeson and others in a study aiming to investigate possible associations between patterns of WM abnormalities and symptom severity in this patient group [129]. In this study, MDD severity was once again correlated with reduced WM integrity in the corpus callosum, but also with reduced integrity in the anterior cingulum, anterior thalamic radiation and sagittal stratum [129]. They also reported that the presence of anhedonia and irritability was associated with disrupted WM tracts in the anterior limb of the internal capsule and in projection fibres to the orbitofrontal cortex and impaired integrity in the anterior corona radiata, sagittal stratum and in efferent tracts leading to temporal and prefrontal cortices [129]. It is also noteworthy that Bessette et al. reported a unique pattern of FA abnormalities in the genu of the corpus callosum, thalamus, midbrain and internal and external capsule tracts, which have yet to be reported in adult-onset MDD [123].

Yuan and fellow workers reported whole-brain reductions in participants with first-episode geriatric MDD in remission, characterised by low FA values in the right superior frontal gyrus, left middle temporal gyrus, left inferior frontal gyrus, right middle occipital gyrus, right inferior parietal lobule, left lingual gyrus, right putamen and right caudate [119]. Taylor et al. also reported widespread decreases in WM FA values in participants with late-onset depression consistent with a state of disconnection between frontal and limbic regions [120]. However, these authors also detected increases in FA values in the frontal gyri and ACC which was predictive of resistance to the selective serotonin re-uptake inhibitor antidepressant sertraline [120]. Interestingly, the same team reported that those with late-onset MDD in remission following antidepressant therapy displayed WM changes which were almost identical to normal patterns of WM integrity seen in never-depressed patients [130].

The weight of evidence indicates that compared with healthy controls, those with midlife-onset MDD display decreased FA values in the left anterior limb of the internal capsule, left posterior cingulate cortex, right cingulate cortex, bilateral parahippocampal gyri, hippocampus, pons, right and left frontal lobes, sagittal striatum and cerebellum [117, 131]. There is also evidence that FA values in the left anterior limb of the internal capsule correlate negatively with MDD severity [117].

Huang and others reported lower FA values in the splenium of the corpus callosum, left cingulum, inferior fronto–occipital fasciculi, superior longitudinal fasciculi and uncinate in patients than in controls, and that this measure might serve as a vulnerability marker for the illness [126]. Xiao et al. reported a virtually identical pattern of WM pathology in the cerebral peduncle, the anterior and posterior limbs of the internal capsule, the external capsule and the cingulum in patients with chronic depression and individuals at high familial risk of developing the illness, suggesting that whole-brain WM disruption precedes the development of active disease [125]. It is also notable that several authors reported significant differences between people with MDD in an acute episode compared with those in remission [132, 133], as well as negative correlations between FA and current illness severity.

Widespread decreases in FA values in the cingulum, superior and inferior longitudinal fascicule and corpus callosum compared with first-episode patients are characteristic of resistant to antidepressant therapy [116]. It is also notable that FA values in the ventromedial prefrontal region in this group are lower even in relation to MDD subjects with a history of remission and recurrence [134]. Finally, recent meta-analyses concluded that compromised WM integrity in the sub-callosal cingulate cortex and in cortical-limbic or cortical-subcortical circuits was most commonly associated with the development of treatment-resistant depression [111, 116].

Not all research teams have reported significant WM abnormalities in MDD patients however [121, 135]. The reasons for such discrepant results are not entirely clear, but differences in technique and evidence that depression is an aetiologically heterogeneous syndrome rather than a single discrete neurobiological entity [136] is likely also relevant.

### Grey Matter Abnormalities in MDD

Several research teams have detected severe or moderate GM volume loss in the amygdala, the thalamus and many cortical brain regions, including the pre- and post-central gyri, orbitofrontal cortex, ACC and temporal cortex and lateral prefrontal cortex [137–140]. Relatively recent meta-analyses presented convergent findings for the ACC and many orbitofrontal cortical structures but divergent findings for limbic and striatal regions [138, 141–143]. Readers interested in a more detailed consideration of the variable findings regarding the patterns of GM pathology in MDD are referred to excellent discussions by [142, 144].

From a treatment perspective, several research teams have reported that patients with treatment-resistant depression display greater reductions in GM volume in the left temporal cortex and right medial frontal cortex compared with age- and sex-matched controls or patients in remission [145, 146]. Reduced GM volume is also a replicated finding in treatment-naïve first-episode adolescent patients [147–149] and indeed may even precede the first episode [143]. The significance of such reductions from a clinical perspective was recently investigated by Luby et al. who reported that the rate of GM loss in adolescent patients correlated with the severity of depression, probably stemming from aggressive synaptic pruning [150].

The pattern of GM structural changes in young first-episode people with MDD is not straightforward, however, with decreases in GM volume in the left lingual gyrus and left putamen and increases in GM volume in the left posterior cingulate cortex and inferior cingulate gyrus, being reported by Yang and others [151]. This abnormal pattern of GM volume change has also been reported by others. For example, Kong and fellow authors reported decreased GM volume in the DLPFC and left frontal gyrus but GM volume increases in the left thalamus and right insula in treatment-naïve patients with first-episode depression [152]. Larger left hippocampal bilateral posterior cingulate volumes appear to be predictive of benefit from antidepressant therapy while low volumes in the hippocampal area in particular are predictive of resistance to antidepressants and disease recurrence. Reductions in hippocampal volume are associated with non-remitting depression, and it is interesting that GM volume change accelerates with duration of illness, concordant with there being an active process of neuroprogression in the disorder [153–155].

### Cerebral Perfusion in MDD

The earliest data were obtained from studies using PET and SPECT [156, 157] based on operator-dependent analysis and revealed a general pattern of reduced regional CBF (rCBF) in the DLPFC, ACC and medial prefrontal cortex [156, 158]. However, the use of techniques such as resting-state perfusion with arterial spin labelling (ASL) perfusion MRI has revealed complex and variable patterns of regional hyperperfusion and hypoperfusion compared with unaffected controls [10, 159–162]. For example, Duhameau et al. reported statistically significant hyperperfusion regions in a depressed patient group compared with a healthy control group in the pallidum, amygdala, putamen, ACC and left prefrontal dorsomedial cortex [161]. On the other hand, another research team using the same imaging techniques combined with whole-brain voxel-based analyses in a study involving medication-naïve adolescent patients and matched healthy controls reported significant hypoperfusion in the amygdala together with paralimbic, frontal and cingulate regions and a pattern of hyperperfusion in the subcallosal cingulate, fusiform gyrus superior insula and putamen [10]. Another imaging technique previously used in cardiac imaging is 320-slice computed-tomography (CT), which was used to assess rCBF in MDD. There was a reduction in the pulsatility index in the MDD group as well as lower rCBF in the left GM than the right in those with MDD [163].

Overall, the most common observations are hyperperfusion in the sub-colossal cingulate [164–166] and hypoperfusion in frontal regions [167, 168], but there is also copious evidence of abnormal perfusion levels in numerous regions of the brain in MDD patients. A recent meta-analysis by Chen and others of studies of resting CBF in medication-free MDD patients obtained by PET, SPECT and ASL revealed elevated values in the right caudate and right thalamus and reduced values in the right ACC relative to healthy individuals [169]. This meta-analysis also revealed somewhat inconsistent changes in rCBF in the frontal–limbic–thalamic–striatal circuits and the precuneus [169].

There are significant differences in the rCBF abnormalities between patients with refractory versus non-refractory MDD ascertained by ASL [161, 164]. One of the largest such studies was of 24 individuals with refractory MDD (RM), 37 patients with non-refractory MDD (NRM) and 42 healthy controls. Individuals with NRM displayed impaired perfusion in the left prefrontal cortex compared with the controls and elevated perfusion largely confined to limbic-striatal areas [164]. RM patients on the other hand displayed impaired perfusion mainly in the bilateral thalamic and frontal regions. Importantly, NRM patients displayed higher CBF in the limbic striatal areas, left and right hippocampal areas and right lentiform nucleus compared to RM patients and healthy controls [164].

Several research teams have also reported an improvement or reversal of rCBF abnormalities following successful antidepressant therapy [162, 170]. For example, Kaichi et al. reported significantly impaired CBF, in the middle and inferior frontal gyri, left inferior temporal gyri and the subgenual anterior cingulate, detected via ASL, which normalised following a 6-week course of the selective serotonin re-uptake inhibitor escitalopram [162]. In addition, Ota and others reported decreased CBF values in the inferior frontal cortex and ACC which reversed following successful antidepressant therapy [170]. This latter finding is of particular interest as there is some evidence that impaired CBF in the ACC and indeed the thalamus may be the cause of the dysfunction seen in MDD patients in these regions of the brain [10]. In support of this view, there are replicated data demonstrating increased glucose metabolism in these areas following successful antidepressant therapy [171, 172] and that the impairment of glucose metabolism seen in active disease may well stem from a decrease in neurones in these brain regions [173].

## Neuroimaging Abnormalities in CFS

### Resting-State Functional Conductivity in CFS

Several authors have reported abnormal patterns of functional connectivity in patients afforded a diagnosis of CFS according to international criteria, whose magnitude correlates with levels of fatigue and pain [174–177]. The precise patterns of impairment are highly variable however.

Wortinger and fellow workers reported a significant decrease in functional connectivity of the salience network to the right posterior insula in adolescent CFS patients compared with age-matched healthy controls. Interestingly, this decrease was negatively associated with the severity of fatigue [174]. In addition, this research team reported a positive association between pain severity and salience network functional connectivity to the left middle insula and caudate which differed between the two groups [174]. Kim et al. also reported an association between altered patterns and strength of functional connectivity and fatigue severity in CFS. In this instance, the authors reported increased resting-state connectivity between the posterior cingulate cortex and the rostral and dorsal ACC whose magnitude significantly correlated with fatigue levels measured by the Chalder Fatigue Scale score while controlling for depression [175]. Gay and fellow workers have also reported widespread impairments in functional connectivity, in this case within the lateral prefrontal cortex and between the occipital lobes and other brain regions [176]. These authors also reported impaired connectivity between the left anterior midcingulate cortex and the sensory motor network and between the left posterior cingulate cortex and the salience network [176]. Importantly, the magnitude of each of these connective impairments correlated with the severity of reported fatigue. On the other hand, Boissoneault and others reported a pattern of increased functional connectivity within and between brain regions in their trial cohort compared with healthy controls. In particular, their CFS participants displayed increased connectivity in the bilateral superior frontal gyrus, ACC, precuneus, thalamus, supplementary motor area, right postcentral gyrus and posterior cingulate gyrus [177]. This contrasted markedly with healthy participants, who displayed greater functional connectivity in the left parahippocampal gyrus, right insula, right precentral gyrus and hippocampus. It is noteworthy however that the relatively reduced level of connectivity in the left parahippocampal gyrus seen in CFS correlated with fatigue severity [177].

This team also examined the patterns of dynamic and static functional connectivity in CFS compared with unaffected controls during a fatiguing cognitive task and reported major group differences. Specifically, Boissoneault and others reported decreased dynamic connectivity between the hippocampus and right superior parietal lobule and increased static inferior frontal gyrus connectivity to occipital and temporal structures and to the cerebellum [178]. It is also noteworthy that healthy controls displayed greater dynamic connectivity increases than did CFS patients between the temporo-occipital regions and insula structures and between thalamus/striatum and the precuneus [178]. Moreover, the degree of static or dynamic connectivity impairment correlated with the self-reported fatigue in CFS [178]. It is difficult to determine a typical pattern of impaired functional connectivity in CFS by a consideration of these studies. It would appear to be different from those displayed in MS and MDD, but clearly, more studies are needed to confirm this, and a study examining resting functional state abnormalities containing CFS, MDD and MS patients allowing direct comparisons to be made could be very informative. Factors likely to affect functional connectivity include peripheral inflammation, disease severity, disease progression and genetic heterogeneity.

### White Matter Abnormalities in CFS

Early research investigating potential neuroanatomical abnormalities using MRI focused on WM using observer-dependent analysis [14, 179–181]. Natelson and others reported subcortical WM hyperintensities in 52 subjects fulfilling the original CDC selection criteria [182] compared with a control group who had undergone MRI investigation for head trauma [179]. On the other hand, Greco and others reported widespread demyelination in many of their CFS participants but at a group level, WM hyperintensities were not significantly different from healthy controls [180]. However, Lange and others also reported significantly increased WM hyperintensities in their CFS group who were free of comorbid anxiety or depression compared with healthy controls and CFS subjects with co-morbid psychopathology [183]. Interestingly, when the data obtained from the two CFS groups were combined, the levels of WM hyperintensities were not significantly different from the control group [183]. In another large study involving 48 CFS patients, Cook and others reported widespread WM abnormalities in their cohort whose extent correlated with symptom severity [14]. It should also be noted that a more recent MRI study using observer-dependent methods failed to detect any WM abnormalities in their CFS cohort although these patients were not recruited according to international consensus guidelines [184].

Recent studies based on T1- and T2-weighted MRI using VBM have universally reported significantly increased WM abnormalities in CFS subjects selected according to the “Fukuda” CDC criteria compared with healthy participants [185–188]. Puri and others reported reduced WM voxel volumes in the left occipital lobe while Barnden and others reported reduced WM voxel volumes in the frontal cortex, caudate and hypothalamus [185, 186]. Pertinently, Shan and others reported decreased WM volume in the left frontal occipital lobe and the fasciculus whose extent increased with self and clinically assessed symptom severity and also worsened over time [188]. Finally, Barnden and others reported reduced WM volume in the prefrontal cortex, the ventrolateral thalamus and internal capsule even following correction for the potential effects of anxiety and depression [187].

### Grey Matter Abnormalities in CFS

Several research teams utilising VBM have reported reduced global or regional GM volumes in those with CFS [185, 188–191]. Puri and fellow workers in a study consisting of 26 CFS participants and 26 age- and sex-matched healthy controls reported significantly reduced GM volume in the left and right occipital poles, left lateral occipital cortex (superior division), left supracalcrine cortex, the posterior division of the left parahippocampal gyrus and the right angular gyrus [185]. Okada and fellow workers on the other hand reported reduced GM volume in the right prefrontal cortex [189]. It is also worthy of note that these authors also reported that the extent of GM volume reduction in the right prefrontal cortex correlated with the severity of reported fatigue [189]. However, it should also be noted that this was a relatively small study consisting of just 16 CFS participants and 16 healthy controls [189].

The results reported by deLange and others appear to be particularly interesting as their studies were specifically designed to examine potential relationships between the severity of symptoms in CFS and cerebral abnormalities and the effect of cognitive-behavioural therapy (CBT) on those cerebral abnormalities respectively [190, 191]. In the first relatively large study consisting of 28 CFS patients and 28 healthy controls, these authors reported significant reductions in global GM volume in CFS and that the extent of such reductions correlated with the degree of physical disability [190]. In the later study, these authors also reported a global reduction in GM volume which increased significantly, albeit slightly (approximately 6%), following a course of CBT [191]. Finally, in their longitudinal MRI study, Shan and others have recently reported a progressive decrease in WM volumes in the left inferior fronto–occipital fasciculus in a cohort of 15 CFS participants (unchanged in 10 normal controls), with decreased regional WM volumes in adjacent regions and GM volume reduction in contralateral regions over a 6-year period; these regional WM and GM volumes once again correlated with illness severity [188].

### Abnormalities in Cerebral Perfusion in CFS

Most of the SPECT studies investigating potential CBF abnormalities in CFS have reported areas of significant cerebral hypoperfusion, at either a global [20, 192] or regional level [193, 194] compared with unaffected controls. However, a minority of researchers reported no significant CBF impairments in comparison with unaffected controls or MDD participants [195, 196]. The reasons for such discrepant findings are unclear, but fewer participants and differences in selection methods may be relevant. The much larger studies conducted by Ichise, Schwartz, Costa and Goldstein, and their colleagues [20, 192–194] appear worthy of particular consideration as they examined multiple brain regions and perhaps even more importantly, apart from Ichise and others [192], examined differences in patterns of abnormal perfusion between participants with CFS and MDD, which is currently a contentious area.

Ischise et al. compared rCBF in 60 CFS participants and 14 healthy controls and reported significant reductions in cortical/cerebellar rCBF ratios in the frontal, temporal, parietal and occipital lobes, providing objective evidence of widespread functional impairment of the brain in the vast majority of their CFS subjects [192]. Schwartz et al. examined SPECT data differences in terms of regional perfusional deficits and mid-cerebral uptake indices between 45 CFS participants, 14 MDD participants, 27 participants with AIDS dementia complex (ADC) and 38 healthy controls [20]. These authors noted more areas of impaired frontal and temporal perfusion in the ADC group compared with those with CFS or MDD. However, the mid-cerebral uptake index was lower in patients with ADC and CFS than in the MDD and healthy controls groups [20].

The largest study examining potential ^99m^Tc-HMPAO SPECT differences between CFS and MDD patients was carried out by Costa and others [193]. It included 40 healthy participants, 67 CFS participants (both with and without psychiatric co-morbidity) and 29 early- and late-onset MDD participants [193]. These authors reported significantly greater global and brain-stem hypoperfusion in CFS participants free of psychiatric symptoms compared with controls and MDD participants, which was not apparent in CFS participants displaying symptoms of anxiety and depression [193]. Goldstein and others compared high-resolution ^99m^Tc-HMPAO SPECT imaging patterns of rCBF in 33 CFS subjects (55 ± 10 years), 26 age-matched MDD participants and 19 healthy participants in an attempt to investigate the potential pathophysiology of CFS [194]. Importantly, they reported dorsofrontal hypoperfusion as the dominant profile in CFS while hypoperfusion seen in MDD was predominantly in the right orbitofrontal lobe, left temporal lobe and, in particular, the left anterior frontal lobes [194].

More recent research using xenon-computed tomography, ASL and high-resolution SPECT reported consistently impaired CBF at a global and regional level at rest and during exercise [197–200]. Yoshiuchi and others reported reduced CBF in the right and left hemisphere in CFS participants free of psychiatric comorbidity compared with healthy controls but also noted that hypoperfusion in CFS patients with psychiatric comorbidity appeared to be exclusively limited to the left hemisphere [198]. These results have been broadly replicated by Biswal and others who reported impaired regional and global CBF in the vast majority of their participants with evidence of regional hyperperfusion in a minority [197]. Evidence of hyperperfusion in some CFS subjects has also been supplied by Machale and others who reported significantly increased hyperperfusion in the left thalamus in CFS compared with a pattern of hyperperfusion in the left prefrontal cortex of MDD [200]. Finally, Patrick-Neary and others reported significantly decreased CBF in CFS participants during maximal exercise compared with healthy controls which they suggested as a cause of the profound disability experienced by many patients [199].

The potential capacity of SPECT to differentiate CFS from MDD may be highly significant as CFS is a purely descriptive diagnosis reliant on phenotype only, and there is accumulating evidence that the diagnosis identifies a clinically and aetiologically heterogeneous syndrome rather than a discrete biological entity even when such a diagnosis is made via the application of international guidelines [1, 3, 201, 202]. Unsurprisingly, research in this area has been bedevilled by a lack of reproducibility which is also the case in many other areas where diagnostic categories are descriptive and homogeneity of causation is assumed (reviewed in [203]). This issue in ‘CFS research’ is even more complex because of the frequent use of unvalidated criteria which only mandate the presence of chronic mental and physical tiredness [204] or where a diagnosis is based on the presence of intermittent symptoms based on a population study of fatigue in a localised population [205]. Hence, a method for increasing the homogeneity of a study population and excluding patients with MDD is to be welcomed. The question of heterogeneity is also relevant to the next section of this paper which briefly examines mechanisms whereby systemic inflammation could be a major element initiating and maintaining neuropathology in different illnesses even though investigation with an array of neuroimaging techniques produces very different and often illness-specific results.

## Factors Affecting Severity and Specificity of Neuropathology

There is widespread agreement that the relationship between the presence of systemic inflammation and various dimensions of neuropathology is established via the conveyance of inflammatory signals to the brain through a range of humoral and neural routes culminating in the activation of microglia and astrocytes [206, 207]. Once activated, these glial cells secrete a range of inflammatory molecules such as pro-inflammatory cytokines, reactive oxygen species, reactive nitrogen species, prostaglandins and quinolinic acid [208, 209]. These are well-documented effects and have been described in a number of detailed reviews [210, 211].

Another source of neuropathology is more indirect however and stems from the loss or corruption of the regulatory functions exerted by these glial cells via a sophisticated communication system with neurones and oligodendrocytes founded on the secretion of exosomes containing mRNAs, miRNAs, caspases and P2X7 (reviewed in [212]). This is a highly complex area, but the key point from the perspective of this paper is that abnormally expressed microglial exosomal miRNAs appear to be independent contributors to the development maintenance and illness-specific patterns of neuroinflammation seen in many different neurodegenerative and neuroprogressive disorders [213]. Thus, microglial activation following prolonged systemic inflammation could produce an illness-specific or at least a range of characteristic WM abnormalities depending on a particular signature of abnormally expressed miRNAs. The mechanisms underpinning this phenomenon are currently unknown but may lie in the recently discovered capacity of at least some miRNAs to activate toll-like receptors and are another mechanism underpinning ‘sterile’ immune activation [214, 215].

Microglia also display distinct regional differences in density and transcriptional identity particularly in bioenergetic and immunoregulatory pathways [216, 217]. This underpins observations of region-specific sensitivities to microglial dysfunction and in particular responses to different pathogen-associated molecular patterns [216, 217]. Hence, this provides another mechanism whereby systemic inflammation could produce geographically variable neuropathology. Another highly pertinent example of the role of microglial genetics in creating unique patterns of abnormalities can be found from recent data in MS, where microglia deficient in the production of caspase-8 are considered to be the source of necroptotic markers seen in active plaques of MS patients. These glial cells are likely one of the main drivers of neuronal and oligodendrocyte destruction seen in this illness [218, 219]. The heterogeneous responses of microglia to external or internal stimuli are also under epigenetic regulation involving DNA methylation, histone acetylation and the expression of a wide range of miRNAs [220]. It is also noteworthy that different genomic patterns of DNA methylation within and outside glial cells are associated with different patterns of neuropathology in distinct brain regions [221].

Predictably, astrocytes also display extensive variability in biochemical, biophysical and immunoregulatory properties in distinct brain regions (reviewed in [222, 223]). The signalling pathways orchestrated by astrocytes are also strongly influenced by genetics [224]. Importantly, some of the neuroprotective functions of astrocytes are also under epigenetic control. Reactive microglia also compromise astrocytic function and survival in an inflammatory environment with the latter property resulting from a decrease of histone acetylation in astrocytes and a silencing of Nrf-2-mediated antioxidant defences [225].

Antioxidant defence systems also demonstrate pronounced regional differences in composition and efficacy. For example, thioredoxin and thioredoxin reductase levels vary in different brain regions [226]. This is also true of glutathione transferase, glutathione reductase and glutathione peroxidase, with the former two enzymes being reduced in the striatum, neocortex, cerebellum and hippocampus, whereas expression of the last enzyme appears to be reduced in the striatum, cerebellum, cortex and corpus callosum [227, 228]. The expression and activity of NADPH oxidases also displays considerable variation in different brain regions [229]. Selenium distribution across different brain regions is also heterogeneous, with the highest concentrations being in the parietal inferior lobule, putamen and occipital cortex and the lowest levels in the cerebellum and medulla [230].

Different discrete neuronal populations existing in different brain regions display differential susceptibility to neurodegenerative stressors which is a phenomenon often described as selective neuronal vulnerability (SNV). Well-documented examples include neurones in the entorhinal cortex, frontal cortex, hippocampal CA1 region and amygdala, which are the most susceptible to neurodegenerative death in populations of neurones most sensitive to the neurodegeneration associated with Alzheimer’s disease [231, 232]. The increased susceptibility of dopaminergic neurones of the substantia nigra to neurodegenerative destruction in Parkinson’s disease is equally well documented [233, 234]. It should be stressed that the pattern and extent of neural damage seen in different neurodegenerative disease is not just dependent on SNV but also stems from specific factors unique to the aetiology of the illness (reviewed in [235]). SNV also exists within populations in the same brain region. For example, the hippocampal CA1 neurones are much more vulnerable than are CA3 neurones to global cerebral ischaemia [236, 237] and oxidative stress [238, 239].

Brain volume and structure are strongly influenced by genetic factors [240–242] and resting-state functional connectivity [243, 244], GM and WM volume [245, 246] and CBF [247, 248] are sensitive to environmentally induced epigenetic changes in gene methylation and/or acetylation and/or changes in the expression of a wide range of miRNAs. These factors may also produce heterogeneity in neuroimaging phenotypes between individuals with the same illness or between illnesses even if systemic inflammation is a major driver of neuropathology in each instance.

## Conclusions and Future Directions

We begin by briefly summarising the key neuroimaging findings.

In MS, there is evidence of impaired neural connectivity and functional dissociation on resting-state fMRI, which correlates with the severity of disability. Cortical demyelination and reduced cerebral perfusion also occur. Early GM atrophy appears to be predictive of the subsequent level of disability and of the prognosis.

MDD is associated with dysfunction of functional connectivity and both microstructural and macroscopic WM abnormalities, including disrupted tracts. There is evidence of GM volume loss in several regions.

CFS is associated with cerebral hypoperfusion, while large VBM studies have shown evidence of GM and WM changes.

The evidence given in this paper points to the likelihood that systemic inflammation may be a major underlying cause of structural and functional neuroimaging abnormalities reported in seemingly diverse disorders including MS, MDD and CFS. Furthermore, reported inter-patient and inter-illness differences may be a function of regional genetic, epigenetic and environmental variations in microglial and astrocytic functioning.

While SPECT appears to be able to differentiate CFS from MDD, its use of ionising radiation makes it unlikely to be a neuroimaging modality of choice in the future for this purpose. Rather, efforts are likely to be undertaken to develop corresponding functional magnetic resonance-based techniques instead. Not only are magnetic resonance techniques free of the use of ionising radiation, they also afford a higher resolution than does SPECT (even high-resolution SPECT). In this vein, a further development is likely to be the use of machine learning classification methodologies, which may, for example, be able in the near future to differentiate different disorders and, within a given disorder, be able to classify patients with a given diagnosis. Finally, there is also a strong case for further combining neuroimaging techniques with investigative modalities from the fields of biochemistry, neurophysiology, environmental medicine, immunology and epigenetics.
